# Management of unusual complications of modified rhomboid/Limberg procedure by interventional radiology guidance; A case report and literature review

**DOI:** 10.1016/j.ijscr.2023.108542

**Published:** 2023-07-25

**Authors:** Athary Saleem, Saqer Alenezi, Ali Alenezi, Omar Alhajri, Fahad Alabdulghani, Ahmed Alkhamis

**Affiliations:** a Department of General Surgery, Al-Adan Hospital, Kuwait; b Department of Radiology, Al-Amiri Hospital, Kuwait; c Department of Surgery, Faculty of Medicine, Kuwait

**Keywords:** Pilonidal sinus disease, Drain fragment, Rhomboid flap, Limberg procedure, Fluoroscopy, Case report

## Abstract

**Background:**

Fragmentation of the surgical drain is an unusual negative consequence of using a drainage system postoperatively. Even though it is rare, multiple management approaches were documented in the literature.

**Case presentation:**

A 19-year-old male patient who had a history of recurrent pilonidal sinus disease that was operated on twice 4 months apart. He presented to our hospital for postoperative follow-up, during which the inserted drain was assessed. While withdrawing the drain, part of it was damaged and missed. The location of the misplaced drain was assessed by a lumbosacral region computed tomography (CT) scan. The decision was made to proceed with less invasive methods using interventional radiology techniques to avoid wide excision and incision reopening complications and prolonged healing time. A fluoroscopy procedure was performed to create three-dimensional anterolateral, frontal posterior views. Then the fragmented drain was retrieved successfully by a minimally invasive technique. The postoperative period was uneventful.

**Discussion:**

Drain fragmentation and/or dislodgement is a highly challenging event that requires highly innovative intervention. Multiple treatment options are available as open surgery techniques and endoscopic approaches.

**Conclusion:**

This case highlights the potential role of fluoroscopy as an outstanding effective choice that could be carried out promptly and safely at the bedside under local anesthetic and reduce the patient's hospital stay.

## Highlights

• Recurrent pilonidal cyst infection commonly requires complex procedures that often require drains.
• Although drain fragmentation is rare, several management approaches were reported in the literature.
• Minimal invasive techniques are effective and safe to treat drain complications.

## 1 Introduction

Surgical drainage systems have been used for both prophylactic and therapeutic purposes as the removal of fluids and blood from surgical sites [1,2]. It is common to use drains postoperatively to avoid fluid accumulation, reducing the risk of infections and anastomotic leak complications [1]. Although surgical draining is common practice, complications are possible [1,3]. Of these complications, the inability to retrieve a surgical drain or its fragment is a rare challenging situation. The underlying etiology is inadvertent suture fixation that creates difficulty during drain removal without tissue damage [4]. Drain fragmentation is possible if the drain is clenched, abnormally strained, or forcefully removed [3]. The prevalence of tethered drains is thought to be between 0.1 % and 0.5 % [4]. The safety and effectiveness of minimally invasive techniques were documented in managing postoperative drain complications [5].

Here we report a case that highlights the potential role of fluoroscopy as a minimally invasive life-saving procedure to manage drain fragmentation that is safely performed for better outcomes, reducing a patient's hospital stay. Our work has been reported in line with the SCARE 2020 criteria [6].

## 2 Case presentation

**Fig. 1:**
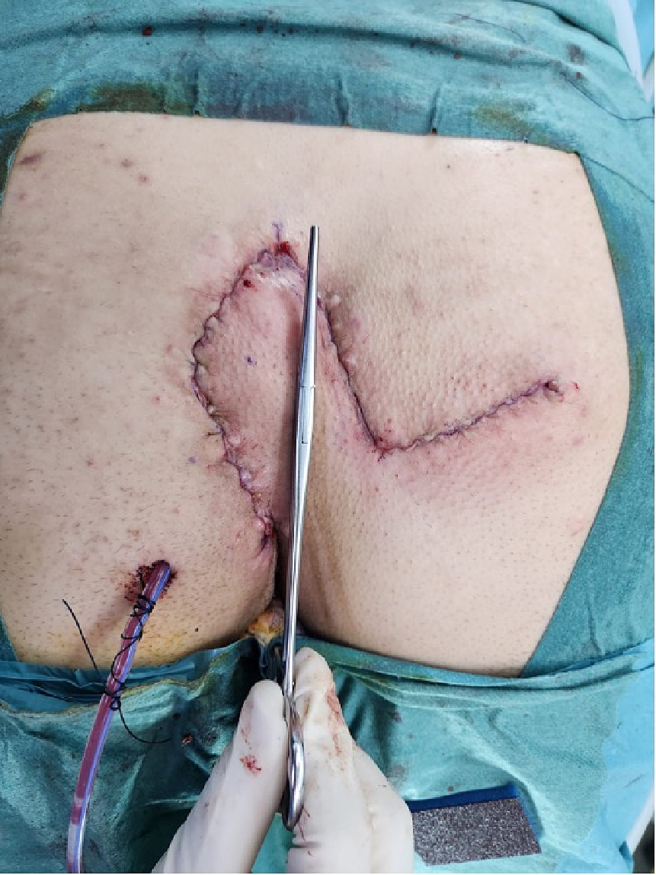
Post-operative figure showing the completed rhomboid flap.

A 19-year-old male patient, presented to our hospital for postoperative follow-up. The patient had a history of pilonidal sinus cyst (PNS) excision with midline closure followed by recurrent PNS infection developed 4 months postoperatively. The recurrent PNS necessitates a modified rhomboid/Limberg flap procedure. During the procedure, 9 cm of PNS-diseased tissue was excised and the wound closed in multiple layers. At the deepest layer a drain was placed (Fig. 1).

**Fig. 2:**
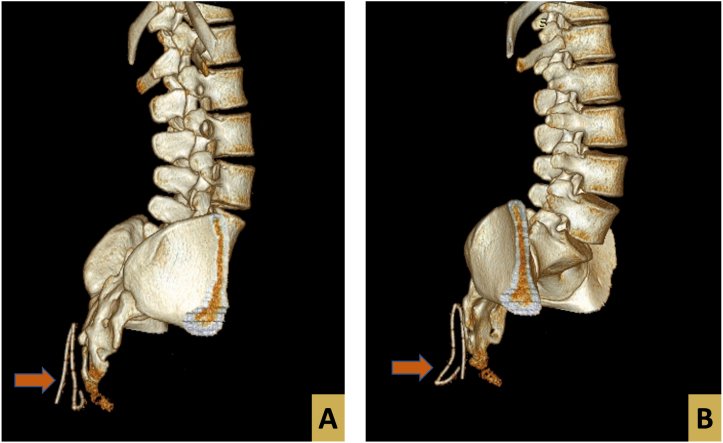
A and B: CT scan showing a three-dimensional view of the lumbosacral region. An arrow illustrates the location of the misplaced drain.

**Fig. 3:**
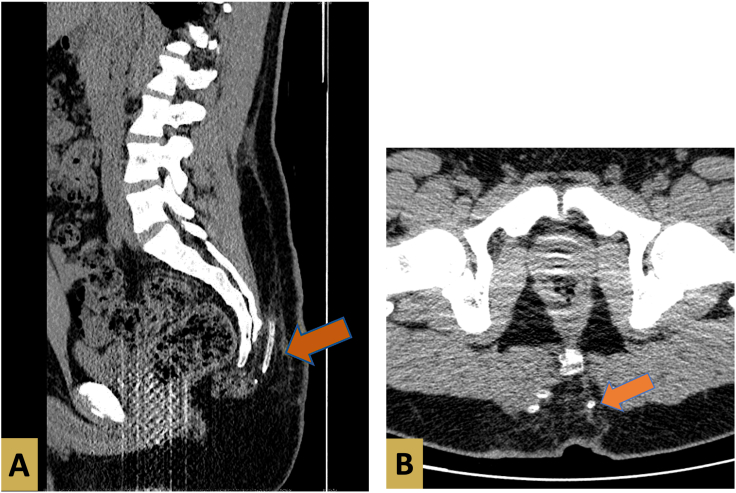
CT scan showing a three-dimensional view of the lumbosacral region. Arrows illustrate the location of the misplaced drain. A: Sagittal view of the CT scan. B: Soft tissue illustration.

The postoperative period was uneventful. While withdrawing the drain on postoperative day 7 part of it was severed and appeared to be missing inside the wound. The position of the misplaced drain was assessed by a computerized tomography (CT) scan of the lumbosacral region as illustrated in the figures below (Fig. 2, Fig. 3).

To avoid opening the wound, potentially increasing the risk of wound complications and prolonged wound healing the decision was made to proceed with less invasive methods by utilizing interventional radiology. A fluoroscopy procedure was performed using multiple X-ray sections, creating an X-ray beam to form three-dimensional anterolateral, frontal posterior views.

**Fig. 4:**
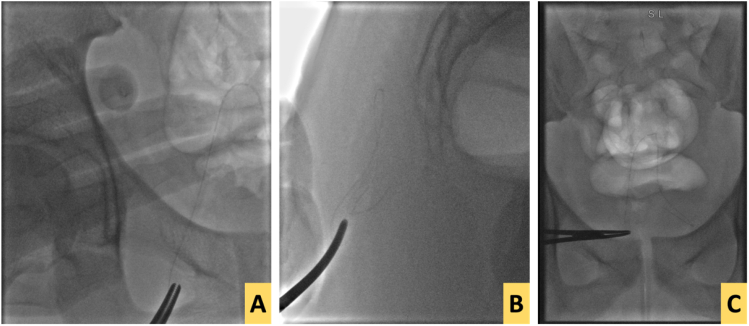
A, B, and C: Fluoroscopy procedure with manual introduction of hemostat arter toward the drain in order to retrieve the fragmented drain.

**Fig. 5:**
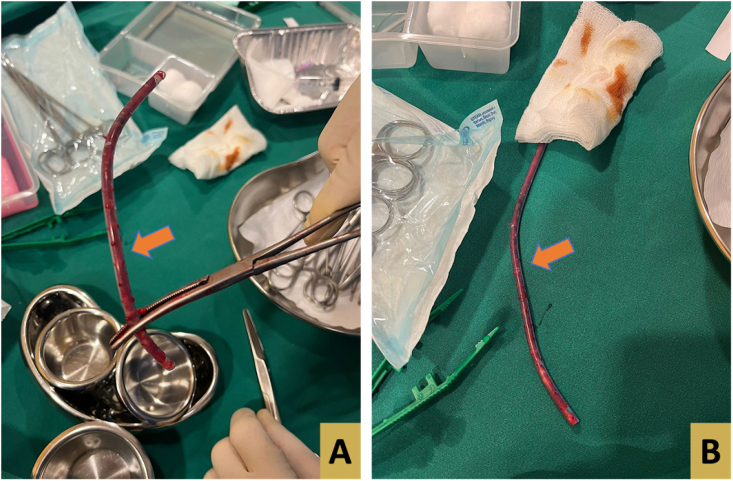
A and B: The withdrawn drain by fluoroscopy procedure.

Once the exact location of the drain fragment was detected, a fluoroscopy procedure was initiated (Fig. 4). After infiltration with wound with local anesthesia the hemostat artery was introduced through the site of drain entry that was located away from the procedure incision site. Under X-ray guidance, further introduction of the artery was achieved manually toward the drain, which was dragged once approached (Fig. 5). the procedure took about 15 min.

The postoperative period was uneventful. This surgical intervention was a rescuing procedure associated with the patient's convenience, allowing him to return to work on postoperative day 14.

## 3 Discussion

Since Hippocrates' day, surgical drainage systems have been used postoperatively as a standard practice. However, there are several complications associated with using surgical drains [1,2]. These include site infections, pain with drain dysfunction, drains twisting and wrapping, vascular injuries of abdominal wall nearby structure perforation, and/or formation of a fistula. Also, an incisional hernia might develop, creating obstructed intestine and incarcerated small bowel loops [1,2].

Generally, there are four etiologies of drain-related complications: (1) improper drain fixation (2) suture material fragmentation (3) reduced intra-abdominal pressure (4) patient's position that may lead to compression on the drain side [1,4]. Also, the incarceration of soft tissue, which is trapped around the drain, and the formation of granulation tissue had a role in drain removal difficulties [4].

The previously published cases documented that the iatrogenic dislodgment of the surgical drain is a challenging situation [3,8,9]. Currently, a number of methods are available to avoid drains from getting tied while being used such as loose drains should be ensured to facilitate its removal before closing the incision [4]. Nevertheless, it occurs frequently for a variety of factors that drains are difficult to eliminate. The main cause of a drain's difficulty to be removed is the unintentional suture placement during the closure, which frequently happens in orthopedic procedures [4,10]. In orthopedic surgical cases, the drain is attached to multiple locations, resulting in retained destroyed drain fragments after an attempt to remove it [10]. Further published research described the relation between the drain that remains attached in multiple places and the occurrence of a high risk of fragmentation [10].

The immediate and accurate identification of the tethered drain location is possibly achieved by ultrasound [4,11]. In our pilonidal cyst wound case, the precise position of the drain fragment was assessed by using a CT scan of the lumbosacral region.

Although drain fragmentation as a rare consequence of surgical procedures is reported in several cases, the optimal management approach remains controversial and needs to be individualized [3,6,13]. The initial method of drain retrieval is to be performed manually [10]. The literature has demonstrated numerous practical approaches for dislodged drain particles [8,9,12]. These include surgical re-operation and endoscopic techniques [8,12,13].

One approach to avoid reoperation and thus potential wound disruption is the use of fluoroscopy and Amplatz sheath in combination with endoscopy for percutaneous retrieval of drains fragments. This approach was reported once, providing effective and safe extraction with minimal tissue injuries in association with the successful handling of foreign intraluminal objects [3].

To the best of our knowledge, our case is the first to report the use of fluoroscopy to retrieve a fragmented surgical drain following a modified rhomboid flap procedure for recurrent PNS. In our case, the drain dislodgement was successfully retrieved using the fluoroscopy procedure with manual manipulation through the lateral drain exit site.

Further studies highlight multiple management approaches for drain fragment retrieval. For instance, a reported case highlights the role of video-assisted thoracoscopy in achieving the successful removal of an iatrogenically retained segment of an intercostal drain, avoiding reoperation [9]. Reoperation and open approaches, as laparotomy, that are utilized to retrieve any retained drain fragments have various disadvantages [1,9,10]. These include increasing patients' exposure to general anesthesia and lengthening their stay in the hospital, enhancing morbidity risk, and adding an additional cost to healthcare facilities [1,9,10].

## 4 Conclusion

Recurrent pilonidal cyst infection commonly requires complex procedures to reduce the risk of recurrence. These include flaps techniques which often require drains. Drains placement can, unfortunately, result in complications such as drain fragmentation and/or dislodgement. In the current situation, our management options were limited due to the location of the fragmented drain and the potential delay in wound healing, and the increased risk of infection if the wound was to be opened to retrieve the drain.

So, a minimally invasive approach was preferable to avoid surgical reoperation to retrieve the drain fragment that was successfully achieved manually aided by the fluoroscopy technique.

## Funding

No funding or grant support.

## Ethical approval

Not applicable.

## Consent

Written informed consent was obtained from the patient to publish this case report and accompanying images. On request, a copy of the written consent is available for review by the Editor-in-Chief of this journal.

## Research registration

Not applicable.

## Provenance and peer review

Not commissioned, externally peer-reviewed.

## CRediT authorship contribution statement

Athary Saleem: literature review, paper writing, editing, picture editing, manuscript drafting.

Saqer Alenezi: literature review, paper writing, and editing.

Ali Alenezi: paper writing and editing.

Omar Alhajri: paper editing.

Fahad Alabdulghani: assisted in surgery, and picture editing.

Ahmed Alkhamis: performed surgery, critical review, paper editing, supervision, and final approval.

## Guarantor

Athary Saleem, D.Pharma., B.Med.Sc., M.D., General surgery department, Al-Adan Hospital, Kuwait.

Email: athary.saleem@outlook.com

## Declaration of competing interest

There are no conflicts of interest to declare by all the authors.
